# The environmental sustainability of data-driven health research: A scoping review

**DOI:** 10.1177/20552076221111297

**Published:** 2022-07-05

**Authors:** Gabrielle Samuel, A.M. Lucassen

**Affiliations:** 1 Department of Global Health and Social Medicine, 4616 King's College London, London, UK; 2 Wellcome Centre for Human Genetics, 6396 Oxford University, Oxford, UK; 3 Clinical ethics, law and society (CELS) Faculty of Medicine, University of Southampton

**Keywords:** Environmental sustainability, environmental impacts, sustainability, digital technologies

## Abstract

Data-Driven and Artificial Intelligence technologies are rapidly changing the way that health research is conducted, including offering new opportunities. This will inevitably have adverse environmental impacts. These include carbon dioxide emissions linked to the energy required to generate and process large amounts of data; the impact on the material environment (in the form of data centres); the unsustainable extraction of minerals for technological components; and e-waste (discarded electronic appliances) disposal. The growth of Data-Driven and Artificial Intelligence technologies means there is now a compelling need to consider these environmental impacts and develop means to mitigate them. Here, we offer a scoping review of how the environmental impacts of data storage and processing during Data-Driven and Artificial Intelligence health-related research are being discussed in the academic literature. Using the UK as a case study, we also offer a review of policies and initiatives that consider the environmental impacts of data storage and processing during Data-Driven and Artificial Intelligence health-related research in the UK. Our findings suggest little engagement with these issues to date. We discuss the implications of this and suggest ways that the Data-Driven and Artificial Intelligence health research sector needs to move to become more environmentally sustainable.

## Introduction

Data-Driven and Artificial Intelligence (DDAI) technologies are rapidly changing the way that health research is conducted.^1^ New technological capabilities to store and process vast quantities of clinical data, and new collections of health data from non-traditional sources,^2^ have led to new opportunities for large-scale data analytics. These in turn have led to the explosion of health data repositories, containing troves of clinical and genomic data,^1^ as well as swaths of self-tracking data from wearables, biosensors and/or environmental data.^3,4^ Meanwhile, social media and other data are being mined for health research,^5^ and machine learning techniques are being used to help predict health conditions.^6^ The storage and processing of health-related data are set to become the fastest growing sector in the datasphere.^7^

Expansion of DDAI technologies inevitably results in adverse environmental impacts. These include heavy carbon dioxide emissions linked to the energy required to generate and process large amounts of data. Approximately 100 megatonnes of carbon dioxide emissions are produced from the digital sector per year,^8^ and the yearly electricity usage of data centres is over 205 TetraWatts per hour,^9^ which already exceeds the consumption of countries such as Ireland and Denmark (see, e.g.^10^) Furthermore, this consumption fails to account for indirect electricity usage (and likely carbon emissions) associated with data centre supply chains.^11^ DDAI technologies also have adverse impacts on the material environment (e.g. *where* data centres are constructed); the unsustainable extraction of minerals for technological components;^8,9^ and e-waste (discarded electronic appliances) disposal.^10^ Rautela and colleagues (2021) note that while rates of e-waste production vary per continent and per capita, as do rates of recycling, in 2019, 53.6 million metric tonnes of e-waste were generated globally (for more information and breakdown, see^12^). Only about one-fifth of e-waste is formally collected and recycled; the fate of the remainder is uncertain, though it is likely disposed in dumps and landfills with other waste, or traded through illegal markets.^13^ These are important concerns: carbon emissions can lead to drastic health impacts^14^ and unsustainable mineral extraction and e-waste practices can have detrimental health consequences – especially affecting individuals in low-and-middle-income countries where mining and waste storage predominantly occurs.

While likely improvements in energy efficiency and the move to renewable energy will no doubt relieve at least some of these concerns,^15^ the pace of data-driven innovation raises concerns that information and communication technologies could outpace the world's renewable energy sources, leading to increases in carbon emissions when other sectors are decreasing their energy use.^11,16^ Furthermore, data-driven solutions have rebound effects, meaning that while digital solutions in the near term may appear to offer environmental advantages, in the long run, this may not be the case. For example, the move to centralise health research data, biobanking data, and/or the move to open science will not *necessarily* lead to less overall reduction in health research data being collected and/or a reduced impact on the environment.

There is a compelling need to consider the environmental impacts of DDAI technologies.^17^ This includes, for example, who is, and who should be responsible for these impacts and how these responsibilities should be enacted and distributed. Given that concern about environmental sustainability in the health research sector is relatively recent,^2^ this paper provides a scoping review to explore how the academic literature is engaging with these issues. Our research question was: what discussions pertaining to the environmental impacts of data storage and processing during DDAI health-related research have been discussed in the academic literature? Using the UK as a case study, we also asked: what policies or initiatives consider the environmental impacts of data storage and processing during DDAI health-related research? Our findings suggest very little engagement with these issues to date.

## Methods

### Literature searches

In October 2021, Web of Science, PubMed and Google Scholar were searched for relevant articles using the keywords in Table 1. Inclusion criteria included documents (book chapters, preprints, conference proceedings, articles, etc.) that focussed on the negative environmental impacts of data storage and processing during DDAI health-related research and care. Health research and care are becoming increasingly interlinked through learning health care systems (see, e.g. the UK's ‘100k Genomics Project’, and ‘Our Future Health’), and widening the search to health care ensured all pertinent articles were included. Relevant exclusion criteria were applied (Table 3). Reference lists of included articles were checked for further relevant documents/authors via snowballing. Following removal of duplicates, 25 documents remained. Six documents – all identified during snowballing – could not be accessed (either because they could not be found in web searches or because they required a fee to access), and so were excluded. The 19 remaining documents were deductively coded into the following categories: title, date published, type of publication (book chapter, journal. etc.), place of publication (journal name, etc.), first author name and country of institution of the first author. The documents were read thoroughly, and additional inductive codes were added, including the type of environmental impact addressed/mentioned; whether the document was framed in terms of green information technology (IT); use of the term ‘sustainability’ as an overarching concept for discussion; and mention of unaddressed challenges and perceived solutions. Documents were also qualitatively reviewed (read in depth) for relevant concepts.

**Table 1.** Search strategy for literature review, including database searched, keystrings used and number of relevant articles retrieved.

Key-string used	Type of article searched	Site searched	Articles searched	Articles deemed relevant
(bioinformatics OR e-health OR m-health OR “health research” OR “health care” OR healthcare OR genom* OR neuroimaging OR radiology OR “medical imaging” OR “electronic health records” OR “health data*” OR “clinical data”) and (digital OR AI OR “big data” OR “big-data” OR “app” OR “tech*” OR “artificial intelligence” OR “machine learning” OR “ICT”) and (sustainab* OR “environment* impact*” OR “environmental* sustainab*” OR “climate change” OR “carbon emissions” OR “e-waste” OR “green”).	Abstract	Web of Science	5172	Following the checking of title, and abstract if needed, 149 which had some relevance. Checking the full article in more detail = 7 of relevance.
-laborator* OR “lab “ OR “labs “ (title) AND “environment* impact” (abstract) -(”lab “ OR “laborator*”)(title) AND green AND “climate change” (abstract) “sustainab* lab*” (title), -"lab “ OR “laborator*” (title) OR environment* OR “sustainab*”	Titles or Abstracts	Web of Science	436	0 articles
(e-health OR m-health OR “health research” OR “health care” OR healthcare OR genom* OR neuroimaging OR radiology OR “medical imaging” OR “electronic health record*” OR “health data*” OR “clinical data”) AND (sustainab* OR “environmental impact*” OR “climate change” OR “pollution” OR “carbon emissions” OR “greenhouse” OR “waste”) AND (digital OR AI OR “big data” OR “big-data” OR “artificial intelligence”)	Titles and Abstracts	PubMed	523	Following the checking of the title, and the abstract/full article if needed, 1 additional article was deemed relevant.
((“lab “[Title/Abstract] OR “laboratory"[Title/Abstract]) AND (“digital"[Title/Abstract] OR “data"[Title/Abstract])) AND (“sustainab*"[Title])	Titles and Abstracts	PubMed	48	0 additional articles
A whole range of keywords with various permutations, for example (“digital health” sustainability “environmental impact”) and (“health database” “environmental impact”) and (e-waste health)	Titles, then abstracts if relevant	Google Scholar	For each keystring, 5 pages of google scholar were checked (in all cases by the fifth page, articles were no longer deemed relevant)	5 additional articles
-sustainab* lab -"science lab” “environmental impact” -Research laborator* environmental impact -Research lab environmental impact -Science lab and environmental impact	Titles, then abstracts if relevant	Google Scholar	For each keystring, 5 pages of google scholar were checked (in all cases by the fifth page, articles were no longer deemed relevant)	0 additional articles
Snowballing				12

**Table 3.** Exclusion criteria for literature searches.

Documents focusing on health and sustainability, or lab sustainability, but not DDAI
Documents that reported appropriate energy sources for wearable biosensors
Documents that related to the positive impacts of data-driven/digital health research or technologies
Documents that explored the environmental impacts of neuroimaging but focused on non-data aspects
Documents that explored power saving in laboratories, where computers were just one component of measures.

DDAI, Data-Driven Artificial Intelligence.

### Web searches

In October 2021, Google was searched using the keywords in Table 2 to identify relevant initiatives and/or policies pertaining to the research question: what policies or initiatives consider the environmental impacts of data storage and processing during DDAI health-related research specifically in the UK? Inclusion criteria included UK web pages associated either with sustainability and the health sector (as above, health care was also included to ensure all relevant initiatives were identified); with research laboratory sustainability more generally; or with sustainability, health sector and DDAI technologies specifically. For each keyword search, all returned pages were checked for at least the first five pages. If at least one relevant link was identified on page 5, the search was continued until two consecutive pages returned no relevant links (the maximum number of pages reached was 10 pages). Exclusion criteria included published journal articles or links to scholars whose work had been identified in the literature review, and multiple links from the same organisations. For each retrieved link (n = 104), information on the weblink's institutional origins, and a short description of the weblink (purpose of the web page; blog, pdf document, policy statement and other information) were collected. All retrieved links were checked for content pertaining to the unintended environmental impacts of data storage and processing for DDAI-associated health research or care.

**Table 2.** List of key strings used to search Google search engine

***Key strings for Google search***
“sustainable lab”
"environmental impact” sustainability health
"environmental impact” sustainability “health research"
"environmental impact” “digital health"
"environmental impact” “digital tech” health
"environmental impact” health “big data"
"health data” sustainability “environmental impact"
"environmental impact” sustainability “health app"
"environmental impact” sustainability health software
sustainability health digital
sustainability “environmental impact” “big data” research
sustainability “environmental impact” AI research
sustainability “environmental impact” digital research
sustainability “environmental impact” “data-driven” research
genomics sustainability “environmental impact"
imaging sustainability “environmental impact”
biobank sustainability “environmental impact"
"electronic medical records” sustainability “environmental impact"
radiology sustainability “environmental impact"
"biometric data” sustainability “environmental impact"
"clinical data” sustainability “environmental impact"
"health data” sustainability “environmental impact"
"genome sequencing” sustainability “environmental impact"
"environmental impact” sustainability health “working group”
"environmental impact” sustainability health “interest group”
"environmentally sustainable” health
environmentally sustainable health society
green “digital health” climate
green digital health climate
green data health climate

### Limitations

While literature and Google searches were kept broad to ensure all relevant articles and UK initiatives/policies were identified, key articles and policies may have been missed. This is because practices pertaining to reducing the environmental impacts of data storage and processing may not be written into policies or initiatives – for example, if they are being considered tacitly and from the ground up. Furthermore, DDAI technology-associated environmental impacts may be being considered as part of broader initiatives that were not detected in our searches. For example, a recent UK Medicines and Healthcare products Regulatory Agency (MHRA) consultation document comprised a section on the environmental sustainability of medical devices that was not detected in our searches. Arguably this was not specifically related to DDAI technologies, but the section highlighted how the health technology sector is beginning to engage with these issues. At the same time as noting these limitations, we emphasise that the aim of a scoping review is to offer insight and summary of an emerging body of scholarship and identify gaps, and to this end, the methodology used is appropriate.

## Findings

### Literature analysis

All analysed documents (n = 19) were published between 2010 and 2021 and included peer-reviewed articles and preprints (n = 7), commentary pieces (n = 3), conference presentations/proceedings (n = 6) and book chapters (n = 3). All conference presentations/proceedings and four articles/commentaries were written for an IT sector audience (n = 10 out of 13, as defined by the journal article or discipline of conference). First, the authors were affiliated with institutions in India (n = 5), the UK (n = 2), Spain (n = 2), South Africa (n = 2) and the United States (n = 2), as well as with institutions in Canada, France, Pakistan, Botswana, Greece and West Africa (n = 1 each). Half of the documents (n = 9) were written by three research groups. Nearly all 18 documents (n = 15) placed their focus on addressing or raising awareness about the environmental impacts of e-health, healthcare and/or hospitals rather than health-related research (n = 3) or health apps (n = 2).^3^

#### Types of environmental impact mentioned

All documents highlighted the importance of reducing energy use and/or carbon emissions to address the environmental impact of digital technologies in the health sector.^4^ Nearly all documents (n = 14) also stressed the need to attend to the environmental impacts associated with the technology's component materials in terms of used mineral resources and e-waste. Scott and colleagues (2012) categorised these environmental impacts collectively into ‘upstream impacts’ (extraction, processing, or synthesis of raw materials, the manufacture of components and the packaging and distribution of these components), ‘mid-stream impacts’ (design, implementation and use) and ‘down-stream impacts’ (‘end-of-life’ aspects of disposal or recycling).^18^

#### Promoting technological solutions

Most documents were technical in nature, meaning that documents focussed on developing *software and hardware solutions* to decrease the environmental impacts of DDAI technologies. Many of the authors developing such solutions defined their approach under the umbrella of green IT (n = 11), that is, an approach to IT that produces minimal waste during its development and operation and promotes recyclability. In fact, green IT approaches were considered by many of the authors as a key approach to addressing the environmental impacts of digital technologies in the health sector.^19^ For example, the authors described their use of green IT to develop efficiency-increasing software, as well as to improve the energy efficiency of data centres serving the health sector. They reported their research on designing the digital infrastructure of smart hospitals^20^ and medical Internet of Things (IoT) systems.^21^ Novel cloud approaches that were more energy efficient were also recommended as solutions.^22^ Saiyeda and Hamdard (2020) provided a roadmap for green IT healthcare that included not only aspects of design and manufacturing, but also purchase, use and disposal of IT technologies.^23^

#### Beyond technical solutions

Few articles considered the *amount and/or type of data collection and processing* as factors associated with the unintended environmental impacts of DDAI technologies in the health sector. One study examined the American College of Radiology (ACR) Appropriateness Criteria for the type of imaging recommended for a specific clinical condition. The authors determined whether imaging modalities used in the US healthcare radiology departments could be switched to those more energy efficient without affecting patient care.^24^ Another separate article raised questions about whether carbon emissions should be a factor in determining the most appropriate video image resolution used during virtual clinical appointments in the UK National Health Service (NHS) because more data is required for higher resolution images leading to a higher environmental.^25^ This same author also described the environmental impacts associated with the exponential increase in data collection and processing in the UK NHS and called for more differentiation between useful and redundant data when considering which data to store: *‘all this data uses energy and infrastructure to store and access. There is a need to start asking the question of whether we need to store all data and how long the data needs to be kept in healthcare settings’.*^25^ Writing in the Lancet, Chevance and colleagues (2020) called for ‘digital temperance’ rather than ‘overconsumption and overpromotion’ of data. These authors described three guiding principles they believed researchers and clinicians should incorporate into their data-relevant practices: restraint in production, use and promotion of digital technologies; lifecycles instead of waste (c.f. the circular economy); and complex systems approaches through interdisciplinary collaboration.^26^

In fact, *collective discussion*, action and responsibility were emphasised by several authors as important for meeting the challenges of environmental impacts. These authors called for health sector workers to collaborate with those in IT, as well as other sectors and industries^23,25,26^ so that together they could properly assess both the state of data use in the health sector, as well as its short-term and long-term direct and indirect impacts. This recommendation has already been followed up, with a group of UK scholars assessing the carbon footprint of bioinformatics in one recent 2021 article.^10^ They found that biobank-scale analyses emitted substantial carbon emissions. These emissions could be reduced by, for example, software upgrades, faster/more efficient computer processing and appropriate data centre choices. Chevance and colleagues (2020) were particularly concerned with rebound effects, that is, where improvements in the technological efficiency of energy use led to greater direct or indirect energy consumption. A need for greater awareness of the environmental impacts of digital technologies in the health sector was stressed.^23,26,27^ Consultation and training on green IT practices (discussed above) were suggested as ways to increase awareness,^27^ as was the creation of standards for assessing and auditing environmental impacts.^19,23,25,26^

#### Sustainability as a normative framework for action

Half of the authors (n = 10) explicitly used the concept of *sustainability* to frame their research. Sustainability was articulated as a valued normative principle and defined as the need to embed economic, social and environmental considerations into the design and use of digital technologies (also referred to in its similar guises of ‘sustainable development’ and ‘the triple bottom line’^5^). Scott and colleagues (2012), for example, developed a responsible response hierarchy for addressing e-waste in e-health^6^.^18^ They argued that respect for the environment is necessary and that ‘*every person or business whose action might impact the environment has an obligation to…act in a manner that maintains a balance between the economy and the ecosystem, and that benefits society at large*’. In some documents, sustainability was defined as being synonymous with green IT (*‘green software often refers to the environmental dimension of software sustainability…In this paper, green software means sustainable software’*).^28^ Several challenges were identified with the implementation of sustainable systems, including cost,^22,23,29,30^ and the need to balance sustainability against other normative values such as privacy, for example, when the local data storage is considered as more privacy enhancing compared to more energy-efficient cloud-based systems.^29^

### Policies and initiatives on the environmental sustainability of DDAI health research or care

Alongside the academic literature scoping review, web searches were conducted to identify relevant policies or initiatives associated with the unintended environmental impacts of DDAI technologies in the UK health research or care sector. Retrieved web pages focusing on laboratory research sustainability (n = 34) or health sector sustainability initiatives more generally (n = 70) were checked for such policies or initiatives. In the former, many institutions and groups have developed resources to assist researchers’ efforts to maintain environmentally sustainable laboratory practices. For example, decreasing the environmental impacts of fume hoods and freezers, reducing water consumption, as well as reducing emissions and waste practices (refuse, recycle, repurpose, reuse and reduce). In these resources, the energy consumption of computers is mentioned, though this is limited to statements concerning the need to turn off digital devices regularly. We were unable to find statements addressing the environmental concerns specifically associated with the unintended environmental consequences associated with DDAI technologies specifically.

In health sector sustainability initiatives, high-level sustainability principles were often presented, with little information about specific practices, policies or initiatives. Because of this, it was sometimes difficult to ascertain how much DDAI-associated environmental impacts were included in these strategies, or whether specific initiatives or policies existed. Several documents found on the searched web pages considered issues associated with the adverse environmental impacts of digital technologies more generally and focused on better assessments of energy use, as well as various practices that could decrease energy consumption. Similar to the sustainable research setting described above, this usually amounted to calls to switch off computers when not in use, or a better estimation of the energy expenditure from digital (and other) technologies. Very few considered the *amount* of data being collected, though, for example, the UK Great Ormand Street Hospital Sustainable Development Management Plan did call for the need to ‘*make visible the emissions for key high-carbon GOSH activities…e.g. data heavy use…*’.^7^ The UK National Institute of Health Research (NIHR) also made reference to avoiding ‘*unnecessary data collection’* in its carbon reduction guidelines.^8^ NHS Digital, which has a specific digital sustainability programme,^9^ discussed the need for *technological* solutions such as low-carbon platforms, consolidating and decommissioning ‘legacy’ data centres and commissioning more efficient data storage. Finally, a Sustainability and Environment Action Special Interest Group formed by the Organization for Human Brain Mapping (OHBM) in 2020 described their engagement with assessing the environmental impacts of neuroimaging DDAI research, and communicating this more broadly to the sector to drive changes in practices. This latter group was the only one we could identify in the UK considering DDAI-specific issues in health-related research.^10^

## Discussion

While the adverse environmental impacts of DDAI-associated technologies are well established, our analysis shows that to date there has been relatively little engagement with them in the academic literature specifically focusing on health, and even less in health research. This might be because most efforts to address the environmental sustainability of DDAI technologies are seen to be an issue for the computing sector rather than as a concern for those who use computational and IT technologies.^31^ It might also be because of current difficulties with calculating the exact environmental impacts of DDAI technologies. For example, there is disagreement and uncertainty in the digital sector about specific carbon emissions associated with different digital technologies, and many of the assessments include varying criteria (e.g. some include embodied carbon or indirect effects, most do not^11^).^32,33^ Furthermore, private corporations are often unwilling to release data necessary for these assessments, requiring the need to develop – at best – rough estimations of such environmental effects. Finally, specific upstream impacts associated with embodied carbon, biodiversity, mining and manufacturing are particularly problematic to calculate because the health research sector only accounts for a small fraction of global use of digital products. Therefore, while steps are being made to assess various environmental impacts of health-related computational research,^10^^12^ much uncertainty remains about how to do so effectively.

At the same time, although environmental impacts are difficult to measure or compare, inaction is not a solution. We need to ‘*shift from treating uncertainty as a temporary issue, as something that more research and more funds can fix, into something that is unavoidably part of science and of policy making’.*^34^ We are left with the question of *how* to consider such impacts. Our findings show that most of the work associated with thinking about the environmental impacts of DDAI technologies in the health sector revolves around providing technological solutions and e-waste management systems through green IT. These are important steps towards addressing many of the issues associated with the unintended environmental impacts of these technologies. At the same time, technical fixes cast situations ‘*as neatly defined problems with definite, computable solutions’,*^35^ masking the complexity of social problems. Such complexity necessitates multidisciplinary approaches that draw on technical fixes, but also on a range of associated social, economic, political and cultural factors. As such – and as some of the authors in our analysis emphasised^23,25,26^ – if we want to consider the environmental impacts of DDAI technologies in health research, we need to look beyond technical fixes to problematise our social practices.^36,37^ The drive to collect and analyse ever-increasing amounts of data is one example.^11^^13^

Problematising ever-increasing data collection and analysis practices requires being attentive to not only the associated potential benefits of these practices, but also reflecting on the economic and political drivers of this ‘datafication’^38^ culture (what are the assumptions that drive us to collect and analyse this data, and where do these assumptions come from), as well as any potential harms that may come from such a culture (including addressing the commonly held misperception that digital technologies do not have an unintended environmental impact). Underlying the ‘more data is better’ culture is the underlying assumption that individuals can be completely knowable if enough data is collected and analysed.^39^ In the health research field, this is seen to lie with more accurate and detailed information that can help improve the health of patients and/or individuals (c.f..^4,35,40^) At the same time, many of these assumptions are tied to economic and political interests: data collection is driven by a cycle of capital accumulation that comes from the expected economic value attributed to the data.^39^ One notable example is genomics, in which the value of health-related data goes beyond health benefits in that genomic technologies attract investments as a way to stimulate the economy towards economic growth.^41^^14^ Coupling genomics and economic growth, while providing much-needed investment in the field, is problematic because it portrays health conditions in technological solutionist frames, amplifies genetic determinism discourses in inappropriate ways, promotes a datafication discourse (if only we had more data our health issues could be addressed; as such store now and if we can think of a use, analyse later), and fuels sociotechnical imaginaries that minimise the complexities of genomic data interpretation.^38,42–44^ While genomics research is vital for a range of specific genetic conditions, coupling genomics with datafication cultures and economic growth also promotes the continued and ever-increasing use of this technology in potentially environmentally unsustainable ways. Datafication in genomics (and health more broadly) also creates a path dependency for digital infrastructures, as well as the epistemic authority that comes from the elevation of certain logics, techniques and imaginaries associated with digital technologies. A sunk cost mentality, that is, the tendency to continue a process after investment and effort, then sees this infrastructure repeatedly built upon.

This does not mean that we should cease collecting and analysing data for health (including genomic) research – especially when analysis could lead to obvious health benefits. Rather, a nuanced approach is required. There is a need to acknowledge that while many health researchers are trying to help the diagnosis, prognosis and/or care of patients and/or individuals with health-related illnesses, their research is embedded within a sociopolitical climate that unquestioningly views datafication as intrinsically good, with little reflection on what this assumption means in practice. Reflecting on this requires those conducting health research to carefully consider the rationale for data collected (who are we helping, but also who (individuals) and what (the environment) are we *not* helping) and refrain from collecting data in such a way that only promotes health and/or societal value for a minority of potential research recipients, or which has no foreseeable value. There is a need to also ensure research *plans* contain pathways and *infrastructures* that allow research impacts to reach wider communities, societies and/or more diverse groups rather than viewing such aspects as a long-term imaginary. This will ensure that while some environmental impacts are unavoidable, research benefits will be as wide ranging and inclusive as possible. To do so, health researchers must consider that many will not be able to afford their potential research outputs, such as drugs or health technologies.^45^ Furthermore, many will not be able to access health benefits because they do not live within the society within which the research is being conducted. These are likely to be those most likely to be harmed by the environmental impacts of DDAI technologies.^15^ Researchers must not be given a ‘free pass’^31^ to defer responsibility away from these issues.

While as stated earlier, it is difficult to understand which choices will have the largest impact on the environment, there are steps that can be taken. At an institutional level, green IT can drive improvements in energy consumption at data centres, e-waste recycling and computer product design and labelling.^46^ At an individual level, several hyperscalers, including Google and AWS (Amazon), now provide carbon emission data to track carbon footprints.^17^,^18^ Other behaviours, such as turning off computers when not in use, can also have an impact on energy use, and various guidelines have been developed to help individuals and institutions align themselves with eco-friendly computing. For example, a University of Cambridge factsheet^19^ notes that leaving a computer on overnight for a year creates enough carbon dioxide to fill a double-decker bus, and while such facts are likely based on contested assumptions and predictions (ref me), they can still be useful for building awareness and driving change. Other helpful guidelines include https://www.greenimpact.org.uk/GIforHealth and LEAF, which is a standard for sustainable laboratory operations.

Lannelongue and colleagues (2021) have proposed a series of 10 rules for health researchers to make computing more environmentally sustainable.^47^ These include calculating the carbon footprint of their research and including this in a cost–benefit analysis; choosing a computing facility and any associated hardware carefully; increasing the efficiency of the code used to analyse the data; and being a frugal analyst during this process (pilot energy-hungry algorithms on smaller data sets first). They also remind researchers to be aware of rebound effects, that is, such as that increases in IT efficiency will lead to increases in demand for data storage and analyses, not a reduction.^48,49^ Taking these rules on board takes seriously the concept of ‘digital temperance’^26^ – it forces us to slow down and think carefully about the specific data we need to collect for our research (rather than collecting as much as possible), and give due consideration to how this data is stored and analysed (c.f. an ‘ethic of care’.^50,51^)

Finally, due consideration should be given to more environmentally sustainable ways of achieving health benefits. This is, of course, not always possible, as we have witnessed during the COVID-19 pandemic, during which decision-making about the most appropriate health interventions has benefited from the collection and analysis of large swaths of data. At the same time, we already know that the biggest improvements to health can be made by addressing its social, economic and political determinants – improving people's lives and well-being (education, economic livelihoods, work-associated stress, etc.), re-balancing economic and social inequality, and improving the quality of our living environments (air and water pollution).

Concluding, health-related DDAI research has tremendous potential to bring health benefits to many, but DDAI technologies also have adverse environmental impacts that must now be addressed.^52^ The health sector has engaged little with the issues that emerge from these impacts. As more and more data are collected for health research – and as more and more societal issues fall under the umbrella of being a health concern and therefore as having an intrinsic value that requires attention (‘healthitisation’^16^ c.f. medicalisation), it is crucial that we explore how to negotiate these challenges. Making explicit decisions about how we do so is the first step to developing shared understandings across the entire research ecosystem about the need to consider these issues within our own research practices. Such work can become a part of the wider sustainability agendas of the UK health sector and/or research institutions.

## Notes

1. For example, UK Biobank, UK national disease registration service, UK health research data alliance; with similar initiatives all over the world.

2. The environmental impacts of DDAI technologies have been known for decades, but have received little attention in the health/research sector until recently (exceptions include, e.g.). Childs S. Editorial: Green Computing. *He@lth Information on the Internet* 2008; 62: 1–2. This is slowly changing, with the need to consider digital impacts comprising aspects of sustainability reports in health. For example, see Health Care without Harm. *Global Road Map for Health Care Decarbonization, Annex C: interventions.* 2021. Health Care without Harm. Furthermore, environmental sustainability has become an increasing concern more generally in health-related academic literature, with special issues being dedicated to this topic. See, for example, https://journals.sagepub.com/topic/collections-hsr/hsr-1-environ_sust/hsrb.

3. One document covered both research and care, which is why the total number of documents is greater than 18.

4. Including issues associated with the excess heat produced during data storage and processing.

5. The ‘triple bottom line’ is a sustainability framework that requires businesses to measure their social and environmental impact alongside their financial profit.

6. Modified from Ahluwalia, Poonam Khanijo & Nema, Arvind K., 2007. “A life cycle based multi-objective optimization model for the management of computer waste” Resources, Conservation & Recycling, Elsevier, vol. 51(4), pp. 792–826.

7. chrome-extension://efaidnbmnnnibpcajpcglclefindmkaj/viewer.html?pdfurl=https%3A%2F%2Fmedia.gosh.nhs.uk%2Fdocuments%2FFOIRQ5888_SDMP_2020_short.pdf&clen=1379688&chunk=true.

8.
https://www.nihr.ac.uk/documents/the-nihr-carbon-reduction-guidelines/21685.

9.
https://digital.nhs.uk/about-nhs-digital/corporate-information-and-documents/sustainability-reports/sustainability-annual-report-2020---2021/content.

10.
https://ohbm-environment.org/.

11. Embodied carbon is the carbon associated with the production of a technology rather than the carbon associated with the technology use. Indirect impacts are non-direct sources of impacts associated with the technology, for example, the environmental impacts associated with e-waste.

12. Also see https://ohbm-environment.org/.

13. This is because Moore's law – which has described an ever-increasing IT system efficiency that has allowed less resources (and emissions) over time for equal efficiency gains – is now slowing down as IT infrastructure reaches its minimal size limits. Rotman D. We’re not prepared for the end of Moore's Law, https://www.technologyreview.com/2020/02/24/905789/were-not-prepared-for-the-end-of-moores-law/ (2020). It is also because technological solutions fail to consider Jevrons’ paradox – that when efficiency is improved it leads to *more* consumption rather than *less* consumption. Alcott B. Jevons' paradox. *Ecological Economics* 2005; 54: 9–21. https://doi.org/10.1016/j.ecolecon.2005.03.020.

14. Also see https://www.gov.uk/government/publications/genome-uk-2021-to-2022-implementation-plan/genome-uk-2021-to-2022-implementation-plan#:∼:text=NHS%20England%20and%20NHS%20Improvement,to%20specific%20groups%20of%20patients and in particular Lord Bethell of Romford's statement, *‘I truly believe that the UK is in a fantastic position to take advantage of our heritage in genomic research and innovation to transform healthcare in this country, to benefit patients and drive our economic recovery’*. This relationship between health and the economy is also evident in other areas, including, for example, stem cell research. See Gardner J, Webster A and Mittra J. The “Entrepreneurial State” and the Leveraging of Life in the Field of Regenerative Medicine. In: V. P and J. G (eds) *Bioeconomies*. Palgrave Macmillan, Cham., 2017.

15. . Finally, this includes minority groups whose data may be removed from analysis to ensure algorithms remain optimised for specific functionality. 

16. Using the term ‘healthitisation’ we mean that increasingly, ever greater forms of data generated in our daily lives (e.g. how we type, how we look at digital screens, how we communicate through digital devices, how we move around social places and in and out of social relationships, etc.) are being framed using health discourses, and as having predictive health value.

17. See https://cloud.google.com/blog/topics/sustainability/reduce-your-cloud-carbon-footprint-with-active-assist and https://aws.amazon.com/blogs/aws/new-customer-carbon-footprint-tool/

18. While cloud computing is often the most environmentally sustainable option, this needs to be balanced against other issues, such as public-private relationships and social justice issues (in terms of hyperscalers approaches to addressing social and environmental concerns).

19. https://www.environment.admin.cam.ac.uk/facts-figures.
